# Electrochemical Assessment of Indigo Carmine Dye in Lithium Metal Polymer Technology

**DOI:** 10.3390/molecules26113079

**Published:** 2021-05-21

**Authors:** Margaud Lécuyer, Marc Deschamps, Dominique Guyomard, Joël Gaubicher, Philippe Poizot

**Affiliations:** 1 Blue*Solutions*, Odet, Ergué Gabéric, CEDEX 9, 29556 Quimper, France; margaud.lecuyer@blue-solutions.fr (M.L.); marc.deschamps@blue-solutions.fr (M.D.); 2 Université de Nantes, CNRS, Institut des Matériaux Jean Rouxel, IMN, 44322 Nantes, France; dominique.guyomard@cnrs-imn.fr

**Keywords:** indigo carmine, solid polymer electrolyte, solid state battery, LMP^®^ technology, organic battery

## Abstract

Lithium metal batteries are inspiring renewed interest in the battery community because the most advanced designs of Li-ion batteries could be on the verge of reaching their theoretical specific energy density values. Among the investigated alternative technologies for electrochemical storage, the all-solid-state Li battery concept based on the implementation of dry solid polymer electrolytes appears as a mature technology not only to power full electric vehicles but also to provide solutions for stationary storage applications. With an effective marketing started in 2011, Blue*Solutions* keeps developing further the so-called lithium metal polymer batteries based on this technology. The present study reports the electrochemical performance of such Li metal batteries involving indigo carmine, a cheap and renewable electroactive non-soluble organic salt, at the positive electrode. Our results demonstrate that this active material was able to reversibly insert two Li at an average potential of ≈2.4 V vs. Li^+^/Li with however, a relatively poor stability upon cycling. Post-mortem analyses revealed the poisoning of the Li electrode by Na upon ion exchange reaction between the Na countercations of indigo carmine and the conducting salt. The use of thinner positive electrodes led to much better capacity retention while enabling the identification of two successive one-electron plateaus.

## 1. Introduction

The year 2021 marks the 30th anniversary of the first commercialization, by the Sony Corporation, of rechargeable lithium-ion batteries (LIBs), paving the way for higher energy density batteries. Their appearance in the world market heralded first a revolution in consumer electronics giving rise to much more flexibility and comfort while making our everyday life easier and safer. In the early 2000s, in the face of global warming and finite fossil-fuel supplies, it was thought possible to consider their integration in other important applications of our energy engineering thanks to the constant and rapid improvement of this technology coupled with potential cost reductions [1]. Today, LIBs support the deployment of decarbonized transportation systems through the massive use of electric motors (the so-called “electromobility” or “e-mobility”) and could enable large-scale storage of electricity produced by the increasingly widespread use of renewable energy sources. Unambiguously, the rise of the Li-ion technology related to the “rocking-chair battery” concept can be perceived as one of the most prominent examples of how chemistry could change our daily life. This fact was highlighted by Zhang et al. [2] in a recent article focused on the history of the LIBs, which honors the pioneer scientists behind it and especially the 2019 Nobel Prize laureates in Chemistry (John B. Goodenough, M. Stanley Whittingham, and Akira Yoshino) as well as M. Armand. Other monographs dealing with this fantastic odyssey are also available in the literature [3,4,5,6,7,8].

In a very simplified point of view, two main pillars have underpinned the development of high energy Li-based batteries: (*i*) the use of the lithium element, which is characterized by low atomic mass and high reducing properties of the metallic state, and (*ii*) the reversible hosting properties of redox-active materials for Li^+^ ions. Starting with the latter, the earliest forms of redox-active insertion materials belong to low-dimensional solids, especially 2-D inorganic structures such as graphite or TiS_2_ (denoted intercalation materials at that time). Seminal contributions on the chemical reduction of such layered materials accompanied with concomitant Li^+^ insertion came from A. Hérold [9] for graphite and W. von Rüdorff [10] and J. Rouxel for TiS_2_ [11,12,13]. Furthermore, in 1972, B. C. H. Steele and M. Armand set the foundations for application of insertion compounds as electrode materials for the electrochemical storage during the *NATO*-sponsored conference held on the shores of Lake Maggiore (Italy) and discussed notably the seminal “rocking-chair battery” concept [14]. Four years later, Steele’s group [15] and S. Whittingham [16] (from the Exxon company at that time) reported simultaneously the electrochemical performance of the first Li-battery based on TiS_2_ as the positive electrode working in a liquid electrolyte medium. On the advice of M. Armand and J. Rouxel, French research and development studies started in 1976 at the Laboratory of the CGE in Marcoussis with A. Le Méhauté, which led to successful collaborative works with the Rouxel’s group in Nantes (including R. Brec, G. Ouvrard, A. Louisy, D. M. Schleich) especially on the study of the LiAl||NiPS_3_ rechargeable battery [17,18,19]. However, LiAl electrodes were not stable enough (rapid passivation and volume expansion during the cycling) while safety issues due to dendrite formation (leading to possible internal short-circuits and thermal runaway) were a major drawback for practical application of Li metal negative electrodes. Following the “rocking-chair battery” concept proposed by M. Armand [14] aiming also at using an insertion material for the negative electrode, in 1980, M. Lazzari and B. Scrosati [20] reported the first Li-ion battery based on the Li*_x_*WO_2_||Li*_y_*TiS_2_ rocking-chair cell assembly. The electrochemical performance of the next LIBs was then widely improved by pairing the layered oxide LiCoO_2_ (developed by the Goodenough’s group in 1980) as the positive electrode with efficient carbonaceous materials (developed by Yoshino and co-workers) on the negative side [4]. Note that a substantial contribution to the good cycling properties of the next commercialized LIBs was achieved with the electrolyte formulation proposed by D. Guyomard and J.-M. Tarascon [21] based on LiPF_6_ as the supporting salt dissolved in ethylene carbonate (EC)/dimethyl carbonate (DMC), which still constitutes the standardized electrolyte formulation of today’s LIBs. Beyond the “rocking-chair battery” technology, which prevents, in principle, the dangerous formation of lithium dendrites at the negative electrode, M. Armand also proposed, in the late 70s, the fabrication of safer Li-batteries by using a dry solid polymer electrolyte (SPE) membrane [22,23]. This SPE membrane is obtained by dissolving a conducting lithium salt in a poly(ethylene) oxide-based matrix (PEO); P. V. Wright and co-workers [24,25] having previously demonstrated that PEO was sufficiently solvating to dissolve alkali salts. Although the formation of Li dendrites was not fully suppressed upon cycling, this SPE membrane can offer an efficient mechanical resistance that is able to protect the positive electrode against short-circuits. However, the best ionic conductivity values in SPE are typically obtained when the amorphous regions of the polymer are favored, which corresponds to temperatures above 60 °C.

After 30 years of intensive research and development to produce ever more efficient LIBs, the current status is that they have probably reached their threshold limit of ≈350 Wh kg^−1^ [26] that falls short of meeting the projected needs in a near future (although it corresponds to a gain of ≈400% in gravimetric energy density compared to the first LIBs produced by the Sony corporation in 1991 [5]). Hence, the all-solid-state battery (SSB) technology based on the implementation of an SPE membrane is inspiring renewed interest in the battery community because it allows the direct use of metallic lithium as the negative electrode. This direction therefore paves the way for developing batteries that are not only safer (free of flammable solvents) but also have a higher energy density [26]. Other kinds of solid-state batteries are intensively investigated by both academic and industrial researchers [27], but the maturity of the all-solid-state batteries fabricated by using a PEO-based solid polymer electrolyte is a great advantage. The Bolloré group and its subsidiary Blue*Solutions* have been developing the so-called lithium metal polymer (LMP^®^) batteries based on SPE since the early 2000s and proved the reliability of this technology in different applications. With the first practical deployment in the world of SSBs in full electric vehicles achieved in 2011, the LMP^®^ technology is now widely used not only in multiple e-mobility scenarios but also in stationary storage applications all over the world (Europe, North America, Asia, and Africa). Like many other electrochemical storage technologies, the search for efficient, cheaper but also earth-abundant insertion electroactive compounds for LMP^®^ batteries remains an ongoing challenge to mitigate their environmental impact [28]. It is now recognized that the use of redox-active organics in batteries, combined with recycling solutions, could decrease the pressure on inorganic compounds and offer valid options to improve the life cycle assessment of cells “from cradle to grave” [29]. Although the volumetric considerations are known to be limiting for organic-based batteries [30], this is not systematically a real issue for some practical LMP^®^ battery applications. Note that the electrochemical performance of *n*-type organic cathode materials in SPE-based SSBs (“*n*-type” electrode reactions involve an ionic compensation with cation release upon oxidation (such as Li^+^) whereas “*p*-type” electrode reactions imply an anion uptake (such as ClO_4_^−^)) has been seldom reported (to the best of our knowledge, by only two studies [31,32]), compared to the hundreds of publications describing organic batteries operating in liquid electrolyte media [33]. However, M. Armand has reported in 1990 the reversible electrochemical accommodation of anions in the *p*-type poly(decaviologen) [34].

**Scheme 1:**
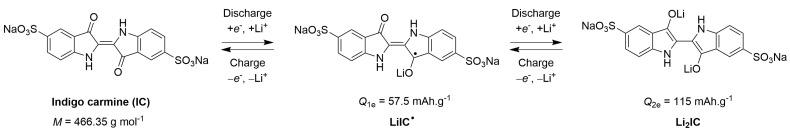
Expected two-electron electrochemical reaction of IC vs. Li.

Considering the potential interest of organic electrode materials for the LMP^®^ technology, the company Blue*Solutions* and IMN joined their know-how to electrochemically investigate selected organic lithium insertion materials. In our first study [31], we planned to test small (neutral) electroactive molecules. Although quite promising in terms of specific capacity, small molecules readily dissolved in conventional liquid electrolytes, ruining rapidly the electrochemical performance. Tetramethoxy-*p*-benzoquinone (TMQ) was chosen due to its unusual but necessary high thermal stability (beyond 100 °C) [31]. Interestingly, we obtained better electrochemical results by using the PEO-based solid polymer electrolyte compared to previous data obtained in liquid electrolyte media, although slow diffusion of the TMQ molecule inside the SPE membrane occurred with time, giving rise to some capacity decay upon cycling. For this second investigation, inspired by our recurrent strategy successfully applied in conventional aprotic liquid electrolytes to circumvent solubilization of organics [33,35,36,37]), we selected an organic salt—namely, disodium 5,5′-indigotin disulfonate, also known as indigo carmine (IC)—as the active material for several reasons. First, it exhibits two permanent and delocalized negative charges in its redox-active organic backbone (Scheme 1), which makes polar interactions with PEO chains difficult.

Second, approved for use as a food colorant, this hydrosoluble synthetic dye is also cheap, renewable, and commercially available at a large scale. Last but not least, IC is already known for its reversible two-lithium insertion process (*Q*_2e_ = 115 mAh g^−1^, Scheme 1) thanks to the electroactivity of its two carbonyl groups at an average potential of ≈2.4 V vs. Li^+^/Li [38,39]. Herein, we report for the first time the electrochemical behavior of IC measured at 100 °C in the LMP technology using a SPE membrane developed by Blue*Solutions*.

## 2. Results and Discussion

**Figure 1:**
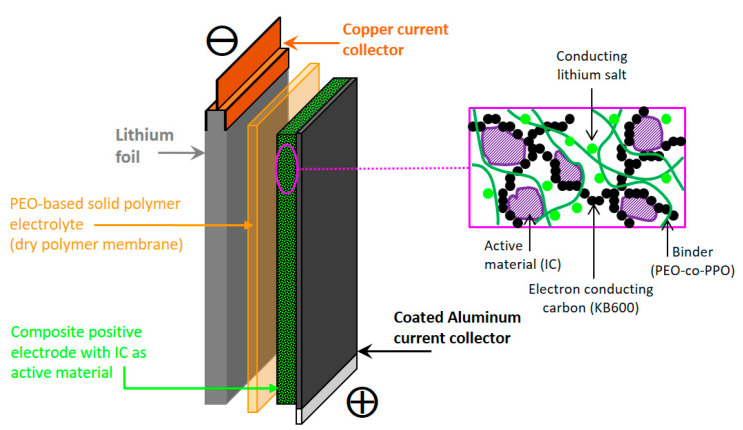
Schematic illustration of an LMP^®^ cell as developed by Blue*Solutions*.

As previously reported [31], the cell assembly used in this second benchmark study on the electrochemical assessment of positive organic electrode materials derived from that presently integrated in LMP^®^ batteries developed and commercialized by Blue*Solutions* since 2011. In short, the all-solid-state lithium organic battery consisted in a simple adaptation of the commercial Blue*Solutions* battery technology using a PEO-based solid polymer electrolyte by replacing the currently used LiFePO_4_ positive electrode material with an active organic material (IC), as illustrated in Figure 1. Hence, results presented in this work are representative of realistic and commercial configurations. All the electrochemical measurements were performed at 100 °C, which is a common temperature for the assessment of the LMP^®^ battery prototypes. Note that IC is thermally stable at more than 200 °C [40], as it is commonly observed in the case of alkali salts of organic compounds [33]. Before presenting the as-obtained results in the LMP^®^ battery technology, we have thought useful to recall the typical electrochemical features of IC-based composite electrodes reported in the literature to date [38,39,40,41].

### 2.1. Recap of the Electrochemical Behavior Concerning Li||IC Cells Measured in Carbonate-Based Liquid Electrolytes at Room Temperature

**Figure 2:**
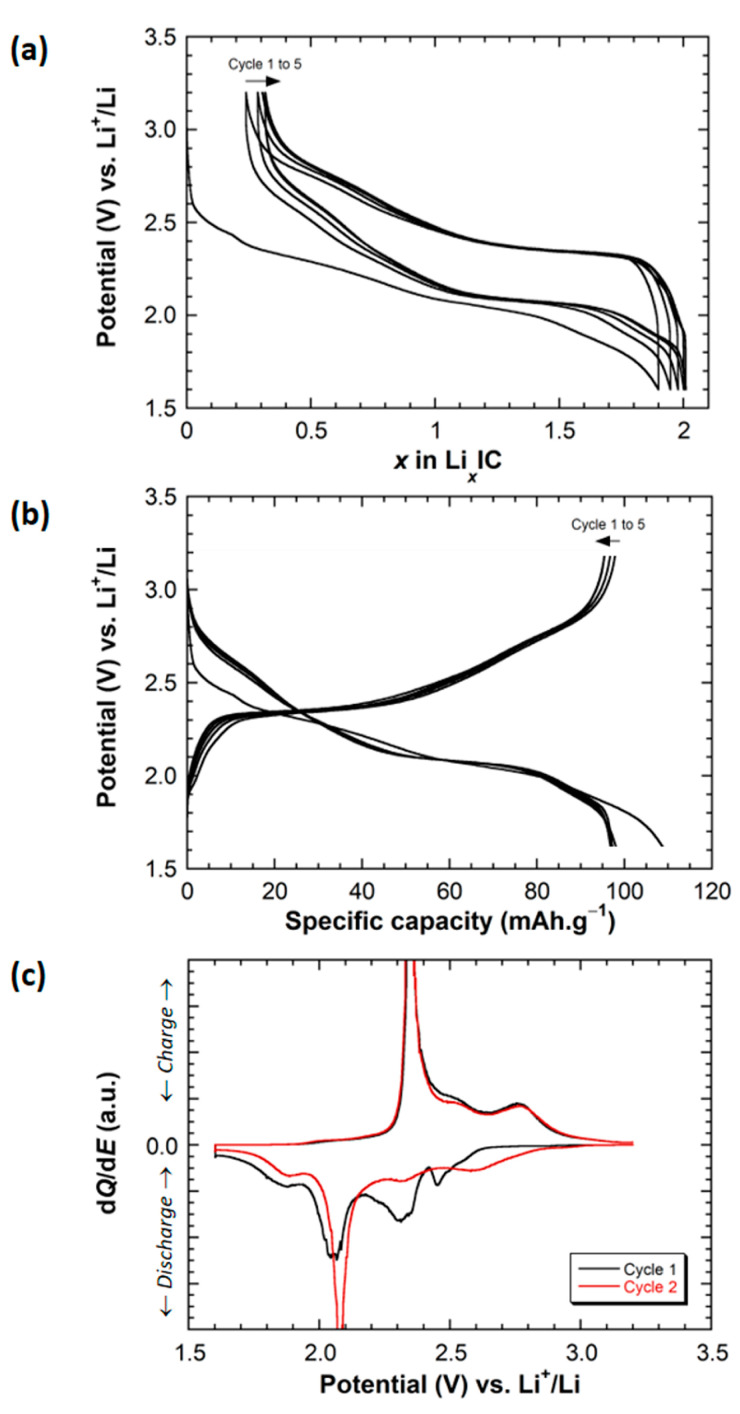
(**a**,**b**) Typical galvanostatic cycling curve of IC measured at 25 °C in Li-half cell configuration for a composite electrode made of IC (85 wt.%) mixed with conductive carbon C-Nergy super C45 (10 wt.%) and carboxymethylcellulose (5 wt.%) as the binder (electrolyte: 1 M LiPF_6_ in EC/DMC; cycling rate: 1 Li^+^ exchanged per mole of IC in 10 h (C/20); electrode thickness: 24 µm) [40]. (**c**) Corresponding differential capacity vs. the potential curve.

Basically, all the reported cycling data were systematically measured in Li- or Na-half cell configuration at 25–30 °C by using carbonate-based liquid electrolytes. Figure 2 shows a representative galvanostatic cycling curve plotted versus both the lithium composition (part a) and the specific capacity (part b) within the 1.6–3.2 V vs. Li^+^/Li potential range. Note that the corresponding composite electrode was specially formulated with only 10 wt.% of conductive carbon thanks to an original aqueous processing previously developed at IMN [40]. During the first discharge, the potential dropped step by step to 1.6 V, giving rise to an overall discharge capacity of 110 mAh g^−1^, which matched the expected two-electron electrode reaction (Scheme 1). The corresponding differential capacity vs. potential curve (Figure 2c) showed the occurrence of four successive steps located at 2.45, 2.30, 2.06, and 1.84 V vs. Li^+^/Li. Upon recharge, most of the inserted lithium ions could be removed of the IC host structure, leading to a reversible capacity above 100 mAh.g^−1^. However, the electrochemical profile differed from the first discharge. First, a plateau occurred at 2.35 V vs. Li^+^/Li, involving half of the reversible capacity, that suggested the occurrence of a first order structural phase transition between the fully reduced compound (Li_2_IC) and the expected radical form (LiIC^•^) based on the description of the electrochemical process at the molecular level (Scheme 1). Afterwards, the potential continuously increased as the delithiation proceeds through two broad peaks (Figure 2c) centered on 2.55 and 2.77 V vs. Li^+^/Li. On the following discharge/charge cycles, these electrochemical features were qualitatively and quantitatively reiterated, giving rise to stable capacity retention. Similar data were reported by the Yao’s group in their seminal electrochemical investigations of IC in Li/Na batteries [38,39].

### 2.2. Electrochemical Behavior of Li||IC Cells Using the LMP^®^ Technology

Our first electrochemical assessment of IC in an LMP^®^ battery was then inspired by our former study performed in a conventional liquid electrolyte medium. Therefore, we aimed at obtaining, for the composite electrode, a similar conductive carbon/IC mass ratio (≈0.14) and a comparable thickness (≈25 µm) although the two electrode formulation technologies were quite different. In practice, the composite electrode was prepared by using carbon black (Ketjenblack EC600JD) as the conductive carbon and LiTFSI as the supporting salt. The same carbon additive was also used in our first organic LMP^®^ batteries based on tetramethoxy-*p*-benzoquinone [31]. The practical details are reported in Section 3.1. The composite electrode was 27 µm thick, its carbon content being 12.5 wt.%.

**Figure 3:**
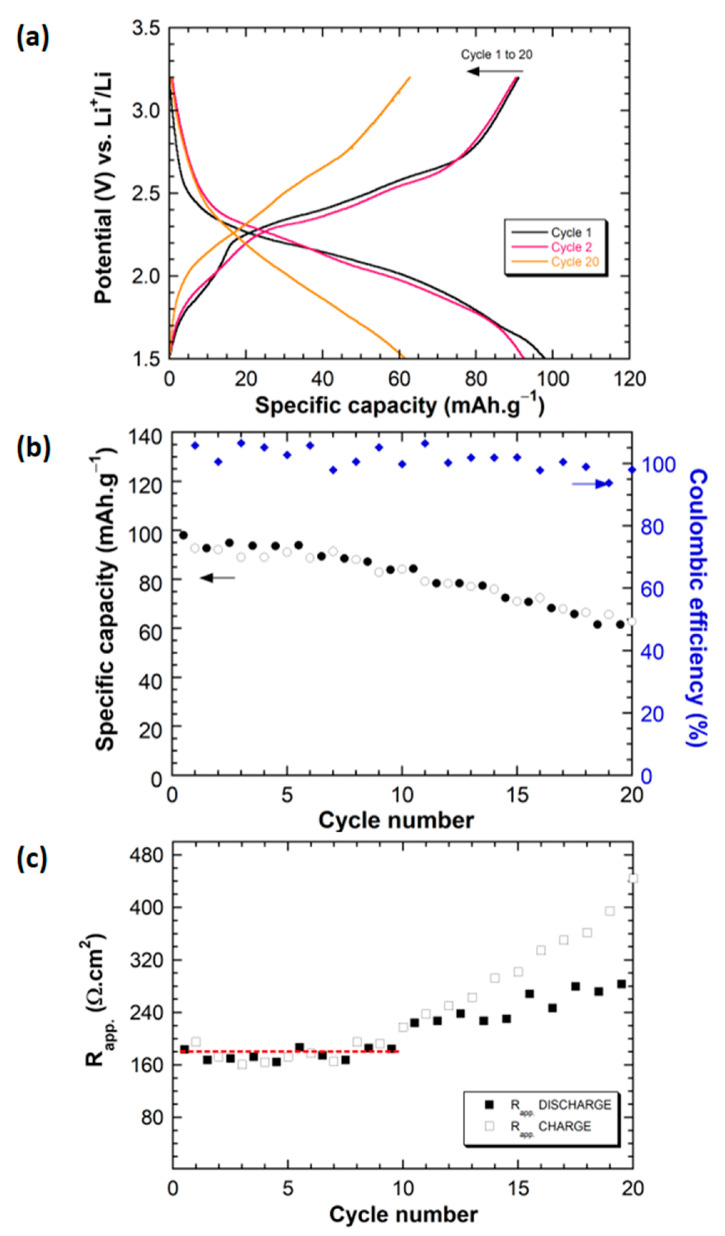
(**a**) Galvanostatic cycling curve evolution (three selected cycles) measured at 100 °C in LMP^®^ cell for a composite electrode made of IC (70 wt.%) mixed with KB600 (10 wt.%), LiTFSI (4 wt.%), and PEO-PPO (16 wt.%); cycling rate: 1 Li^+^ exchanged per mole of IC in 2 h (C/4); electrode thickness: 27 µm. (**b**) Corresponding capacity retention curve together with the coulombic efficiency. (**c**) Evolution of the apparent cell resistance, both on charge and discharge, upon cycling. The red dashed line highlights the relative stability of R_app._ during the first ten cycles.

Unfortunately, as opposed to performance reported in the literature using conventional carbonate-based liquid electrolytes, the as-obtained electrochemical results did not meet our expectations (Figure 3). More precisely, the representative galvanostatic cycling curve of such LMP^®^ cells (Figure 3a) plotted within the 3.2–1.5 V vs. Li^+^/Li potential window showed an obvious capacity fading coupled with an increase of the cell polarization upon cycling. Figure 3b allows a better visualization of this trend with an irregular but continuous capacity decay of about 40% over the 20 first cycles, when the coulombic efficiency was instable. The corresponding evolution of the apparent cell resistance (R_app._) provided complementary and useful information (Figure 3c). After a period of relative stability at ≈180 Ω.cm^2^, a sharp resistance increase could be noticed after 10 cycles, especially in charge reaching more than 440 Ω.cm^2^ at the 20th cycle. As the polyanionic nature of IC should have prevented the possible dissolution/diffusion within the SPE, as explained above, such an electrochemical behavior pointed to the occurrence of interfacial issues that worsened with operating time. Let us recall that such poor electrochemical features were not observed when using the neutral TMQ molecule as the positive active material in LMP^®^ cells despite its slow diffusion within the PEO-based network [31]. In this trial, despite the poor cycling stability of the cells, the electrochemical behavior was consistent with previous results, either already published in the literature or obtained in carbonate-based electrolytes. Basically, the shape of the electrochemical curve recorded during the first galvanostatic cycle remained roughly similar to that reported in conventional carbonate-based liquid electrolytes, which confirmed the reversible electroactivity of IC vs. Li at 100 °C in the LMP^®^ cell.

**Figure 4:**
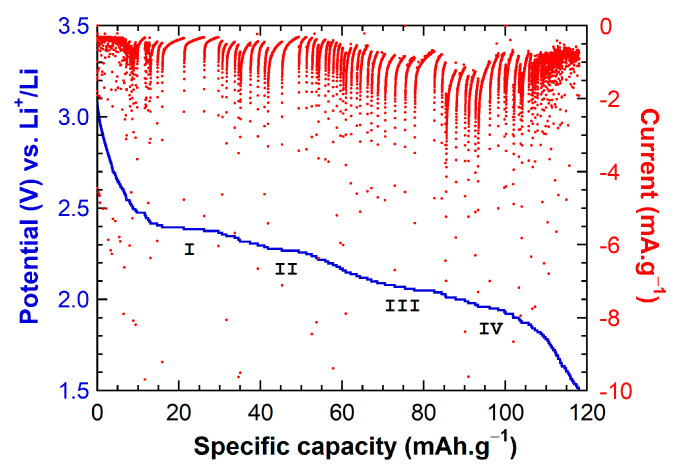
PITT measurements at 100 °C in an LMP^®^ cell for a composite electrode made of IC (70 wt.%) mixed with KB600 (10 wt.%), LiTFSI (4 wt.%), and PEO-PPO (16 wt.%); electrode thickness: 48 µm; Δ*E* = 10 mV with a current limitation corresponding to C/400 (i.e., *i*_min_ = 0.29 mA g^−1^).

Thicker IC-based electrodes (48 µm) were then prepared and electrochemically tested in similar cycling conditions, but we experienced faster loss in the cycling stability with rapid increase of the cell polarization. This second series of experiments was an occasion to probe more accurately the electrochemical lithium insertion processes into IC by running a potential-controlled mode: potentiodynamic intermittent titration technique (PITT). An advantage of the PITT method is that the electrochemically driven phase transitions in insertion electrodes can be more readily visualized by analyzing both the potential evolution and the corresponding current response [42,43]. Figure 4 shows the as-obtained potential/current profiles recorded during the first discharge. Despite the thickness of the electrode, the potential trace reveals the existence of four main steps (pseudo-plateaus) located at 2.39, 2.30, 2.06, and 1.96 V vs. Li^+^/Li, which are in very good agreement with the values reported by using conventional carbonate-based liquid electrolytes (Figure 2). Those pseudo-plateaus are correlated with bell-shape-type responses in current (non-Cottrellian-type decay) revealing four successive phase transformations (I to IV). Interestingly, one can notice a larger potential gap (i.e., energy gap) between steps II and III at half of the reversible capacity, which formally corresponds to the formation of the expected radical form (LiIC^•^), as underlined above.

**Figure 5:**
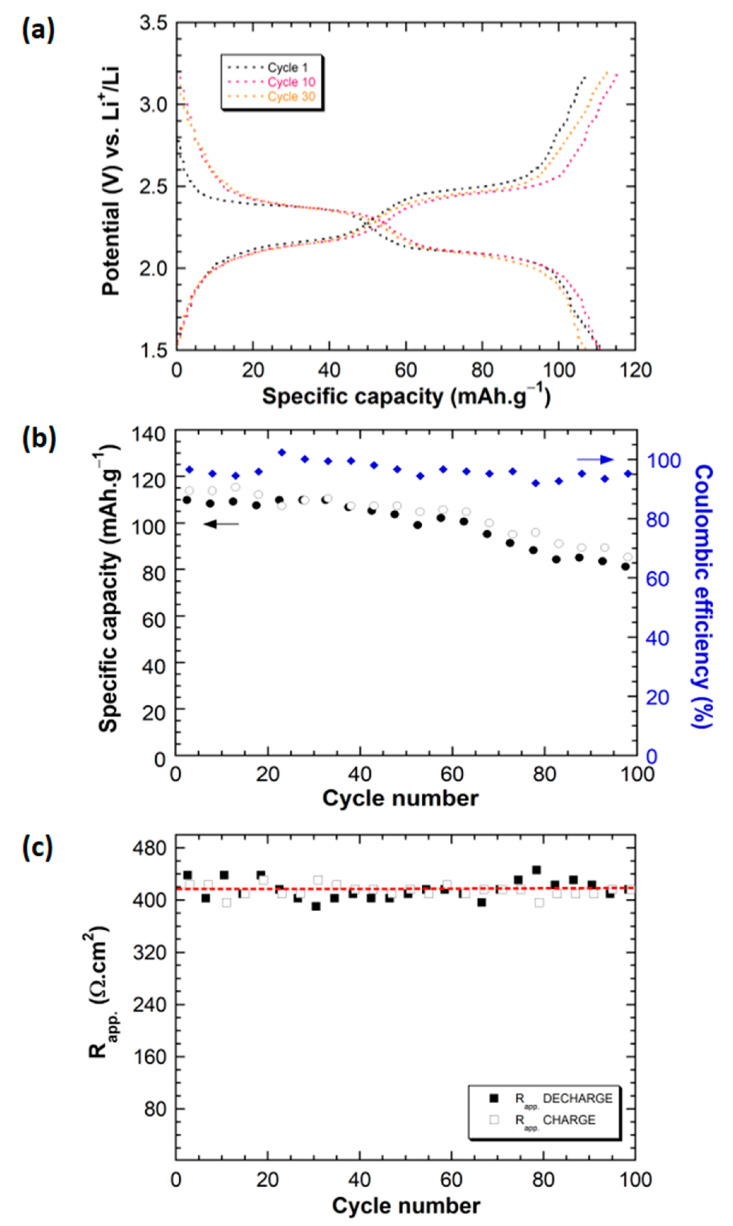
(**a**) Galvanostatic cycling curve evolution (three selected cycles) measured at 100 °C in LMP^®^ cell for a thin composite electrode made of IC (70 wt.%) mixed with KB600 (10 wt.%), LiTFSI (4 wt.%), and PEO-PPO (16 wt.%); cycling rate: 1 Li^+^ exchanged per mole of IC in 1 h (C/2); electrode thickness: 6 µm. (**b**) Corresponding capacity retention curve together with the coulombic efficiency. (**c**) Evolution of the apparent cell resistance, both on charge and discharge, upon cycling. The red dashed line highlights the relative stability of R_app._ upon 100 cycles.

Thinner IC-based electrodes (6 µm) were also fabricated and electrochemically tested to complete our screening. To our surprise, the as-obtained electrochemical behavior was very different compared to previous data. An example of a galvanostatic cycling curve recorded at C/2 is shown in Figure 5a. The first striking feature was the drastic simplification of the potential-capacity trace. First, the four pseudo-plateaus conventionally observed in the first discharge merged, giving rise to two successive and well-defined plateaus, fully reversible, and corresponding to the storing of one *e*^−^/Li^+^ per IC each. The two characteristic average potential values can be determined at 2.43 and 2.11 V vs. Li^+^/Li. These two successive one-electron electrode reactions are well aligned with the molecular-level description of the electrochemical process (Scheme 1) confirming unambiguously the particular stability of the radical LiIC^•^ phase in the solid state, in agreement with the PITT measurements using a thick electrode (Figure 4) and the electrochemical data obtained in organic liquid electrolyte media [38,39,40]. This thin electrode was able to sustain its capacity over dozens of cycles, as shown in Figure 5b. Nevertheless, a capacity decay became visible after 60 cycles (≈25% of loss after 100 cycles). The corresponding evolution of the apparent cell resistance (Figure 5c) was certainly much more constant, on average, than that observed with the 27 μm thick electrode, but a certain instability remained observable. In short, these characteristics confirmed again some interfacial limitations, even if the use of a thin electrode enables much better stability on cycling and a markedly improved electrode kinetics.

### 2.3. Post-Mortem Analyses and Failure Identification

**Figure 6:**
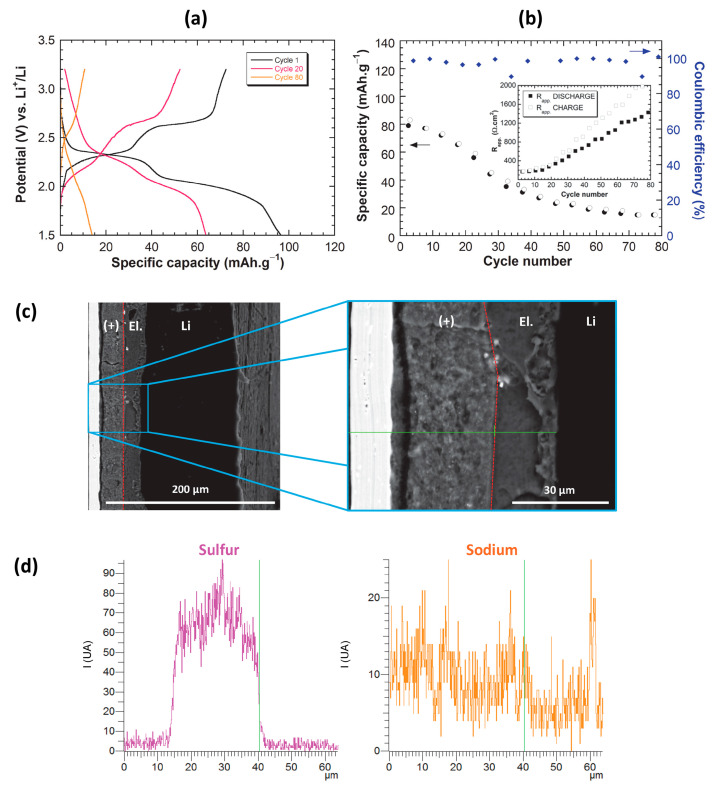
Post-mortem investigations of a LMP^®^ cell stopped after 80 cycles for a composite electrode made of IC (70 wt.%) mixed with KB600 (10 wt.%), LiClO_4_ (2 wt.%), and PEO-PPO (18%); electrode thickness: 23 µm. (**a**) Galvanostatic cycling curve evolution of the cell (cycling rate: 1 Li^+^ exchanged per mole of IC in 1 h (C/2)). (**b**) Corresponding capacity retention curve together with the coulombic efficiency. Inset, evolution of the apparent cell resistance upon cycling. (**c**) SEM imaging (edge view) of the full cell together with a magnification of a representative part of the current collector/composite electrode/electrolyte surface area. The red dashed line indicates the boundary between the positive electrode and the electrolyte; (+): IC-based composite electrode; **El.**: solid polymer electrolyte; **Li**: metallic lithium for the negative electrode. (**d**) EDS spectroscopy results (line profile along the green line shown in (**c**)) highlighting the presence of sodium traces through the full thickness of the cell and more especially at the Li interface. The vertical green line corresponds to the boundary between the composite electrode and the SPE layer.

In order to better understand what could hinder the proper functioning of IC as an active electrode material in LMP^®^ cell, especially when increasing the electrode loading, post-mortem investigations were performed by coupling SEM imaging and EDS analysis (see details in Section 3.2). During the preparation of the sample for SEM/EDS characterizations, we did not notice any change of the pristine colorless electrolyte film (IC being a blue dye), contrary to our former observations with TMQ as the active material; the typical orange color of TMQ was indeed clearly visible to the naked eye in the SPE layer [31]. However, the simultaneous presence of sulfur atoms, both in the IC chemical structure and in the conducting salt (LiTFSI), complicated the interpretations of the EDS measurements. To avoid this interference, a new series of LMP^®^ cells was prepared by using LiClO_4_ as the conducting salt. Note that the fabrication of relatively thick composite electrodes was preferred for this investigation to make the SEM/EDS characterizations easier and because rapid capacity decay was anticipated. Figure 6a,b shows the corresponding electrochemical cycling data. As expected, the reversible capacity drops rapidly upon cycling, recovering less than 20 mAh g^−1^ after 80 cycles. In addition, the peculiar sharp increase of R_app_. was also noticed after 10 cycles, in agreement with the data reported in Figure 3c where LiTFSI was used as the conducting salt. However, the galvanostatic trace revealed more clearly the occurrence of two successive plateaus. Figure 6c,d summarizes the main characteristics obtained from such post-mortem studies. Interestingly, no obvious degradation of the components of the LMP^®^ cell was observed by SEM after cycling (Figure 6a). Moreover, the sulfur content being close to zero in the electrolyte compartment, it was well confirmed that no diffusion of the IC dye occurred upon cycling through the cell, in accordance with the macroscopic observation (to the naked eye) of the SPE layer and our initial expectations based on the polyanionic nature of the IC backbone that would impede polar interactions with the PEO chains. However, the EDS line profile pointed out the presence of sodium traces through the full thickness of the cell with an obvious accumulation at the lithium interface. This important result reveals that a Na/Li exchange reaction occurred between the active material and the lithiated conducting salt; Na^+^ ions being subsequently transported by the PEO matrix and reduced at the Li negative electrode. The contamination of the reactive lithium interface by Na supports well the interfacial perturbations observed during the electrochemical measurements, especially the rapid increase of the apparent cell resistance. Indeed, the magnification by the SEM, shown in Figure 6c, revealed some damages at the electrolyte/Li interface making it more resistive. Consequently, one could easily understand that such perturbations were found to be more pronounced with the operation time and for higher electrode loadings (i.e., when more Na atoms were present inside the cell). This possibility of ion exchange reaction between these two alkali metal ions could have been anticipated because the Na^+^ mobility in PEO matrix had been demonstrated by the pioneer work of P. V. Wright and co-workers [24] but not necessarily this marked instability of the electrochemical interfaces. Therefore, the promising cycling data obtained when using thin IC-based composite electrodes confirmed the stable reversible electrochemical activity of the sodium (*E*)-3,3′-dioxo-[2,2′-biindolinylidene]-5,5′-disulfonate redox center and thus it can be anticipated that replacing IC with the corresponding lithiated salt should allow better electrochemical performance in LMP^®^ technology (note this salt is not, however, commercially available).

## 3. Materials and Methods

### 3.1. Reagents, Electrode Preparation, and LMP^®^ Cell Assembly

Indigo carmine (Sigma Aldrich, St. Louis, MO, USA, for microscopy), lithium bis(trifluoromethanesulfonyl)imide (LiTFSI, 3M), tri-hydrated lithium perchlorate LiClO_4_·3H_2_O (Aldrich), carbon black Ketjenblack^®^ EC600JD (denoted KB600, AkzoNobel), poly(ethylene oxide)/poly(propylene oxide) copolymer (denoted PEO-co-PBO or ICPSEB, Nippon Shokubaï) were used as received. The composite electrode was prepared as follows: An amount of 4 g of carbon black KB600, 1.6 g of LiTFSI, 6.4 g of ICPSEB (binder), and 28 g of IC were mixed together in water and introduced in a plastograph^®^ Brabender^®^ and maintained at 80 °C at a rotation speed of 80 rpm for 20 min. The as-obtained paste was spread between two heating-rolls (95 °C) over an aluminum current-collector coated with carbon. Then, water was evaporated in an oven for 20 min at 105 °C in a dry room with controlled moisture (dew point of −55 °C). Two representative electrode thicknesses were more specifically selected: 27 µm (0.30 mAh cm^−2^) and 6 µm (0.07 mAh cm^−2^). Note that 0.81 g of LiClO_4_·3H_2_O and 7.55 g of ICPSEB were used in the case of composite electrodes containing LiClO_4_ as the conducting salt (LiTFSI-free composite electrodes, thickness: 23 µm). Lithium foils for the negative electrode as well as PEO-based electrolyte membranes (O/Li ratio = 25) were provided by the Blue*Solutions* company. The polymer cells were assembled in a dry room by stacking lithium, solid polymer electrolyte membrane, and the composite IC-based positive electrode onto its current collector at 80 °C. On the negative side, the current collector was a copper foil hand-welded to lithium. Each current-collector was assembled to copper-connectors by spot welding. Cells were air tight sealed in coffee-bags and vacuumed in order to avoid air pockets (geometrical surface area: ≈20 cm^2^).

### 3.2. Electrochemical Measurements and Characterization Techniques

The galvanostatic cycling data of the cells were measured by using a MCV 64-1/0.1/0.01/-5 CE battery testing equipment (Bitrode). The apparent cell resistance (R_app._), also known as internal cell resistance, was determined by the simple current interrupt technique by using the open circuit voltage (OCV) periods integrated in the cycling program at each half-cycle. A VMP3 system (Bio-Logic SAS, Seyssinet-Pariset, France) was used for the PITT measurements. The reported capacity values referred to the mass of the active material (IC) in the positive electrode. For the post-mortem study, the cell was opened in a dry room. A 1 cm^2^ piece of this cell was cut with a blade, frozen in liquid nitrogen, and finally sliced with a microtome to get proper cross-sections. Morphology of the as-prepared samples was then investigated by scanning electron microscopy (SEM) using a Hitachi TM3000 equipped with a Bruker Quantax 70 for energy-dispersive X-ray spectroscopy (EDS) analysis.

## 4. Conclusions

The electrochemical behavior of IC was described for the first time in an SPE-based SSB using the LMP^®^ technology commercialized by Blue*Solutions* at an operating temperature of 100 °C and for a low conductive carbon/IC mass ratio (≈0.14). First, ≈25 µm thick electrodes were evaluated to be compared with the cycling data, reported in the literature, obtained at 25–30 °C in Li-half cell configuration by using carbonate-based liquid electrolytes. The as-obtained galvanostatic cycling curves were well aligned with the expected electrochemical features associated with the occurrence of a reversible two-electron process. However, the cycling performances were altered as a result of interfacial issues at the lithium negative electrode. The latter stemmed from the presence of sodium upon the Na^+^/Li^+^ ion exchange between the SPE electrolyte and IC. Much better capacity retention curves were obtained when we decreased the Na contamination thanks to the use of thinner electrodes (6 µm). For such electrodes, the galvanostatic cycling profile was, in addition, significantly modified and exhibited two fully reversible and well-defined plateaus, corresponding to the storing of one *e*^−^/Li^+^ per IC in line with the description of the electrochemical process at the molecular level. This investigation therefore reinforces the interest of using organic salts (rather than neutral molecules) as active materials to suppress the possible solubilization/diffusion into the SPE membrane and highlights that caution should be taken to avoid alkaline ions mixing within a Li cell.

## Data Availability

Data are the property of Blue*Solutions*.

## Sample Availability

Samples of the compounds are not available from the authors.
